# Supplementary material for: Evaluation of Aedes aegypti control intervention with pyriproxyfen by lcWGS in Manacapuru, Amazonas, Brazil
Source: PLoS Negl Trop Dis. 2024 Oct 3;18(10):e0012547. doi: 10.1371/journal.pntd.0012547 (PMC11478823; doi:10.1371/journal.pntd.0012547)
Supplement: Attachment — Submitted filename: Cover_letter_260624revisores.docx [file pntd.0012547.s002.docx]

**Reviewer's Responses to Questions**

**Key Review Criteria Required for Acceptance?**

**As you describe the new analyses required for acceptance, please consider the following:

Methods**

- **Are the objectives of the study clearly articulated with a clear testable hypothesis stated?**
- **Is the study design appropriate to address the stated objectives?**
- **Is the population clearly described and appropriate for the hypothesis being tested?**
- **Is the sample size sufficient to ensure adequate power to address the hypothesis being tested?**
- **Were correct statistical analysis used to support conclusions?**
- **Are there concerns about ethical or regulatory requirements being met?**

**Reviewer #1: Objective of the study needs to be reframed as it is not apparent to the reader.**

A: We appreciate your suggestion. We have taken your feedback into account, reformulated the objective to make it clearer and more evident to the readers, and highlighted it in the revised text. We believe that this modification will enhance the understanding of the study and its relevance.

**Reviewer #2: Yes the methods are coeherent with the objectives and are well described to allow to replicate the experiments. Sample size is fine and appropriate to test initial hypothesis. there is no concern about ethical and/or regulatory issues.**

A: We would like to express our sincere gratitude for the positive comments regarding our manuscript. We are pleased to know that methods and sample size were considered coherent, and the objectives well described, allowing the replication of the experiments. We are committed to addressing the suggestions made and ensuring that our study significantly contributes to the advancement of knowledge in the field.

**Results**

- **Does the analysis presented match the analysis plan?**
- **Are the results clearly and completely presented?**
- **Are the figures (Tables, Images) of sufficient quality for clarity?**

**Reviewer #1: The captions for Figure 4 and 5 need to include additional information to enhance clarity. Terms such as 'Post' and 'Pre' should be briefly explained within the captions to provide context for the reader.**

A: We have revised the captions for figures 4 and 5 to include the additional information and brief explanations of the terms 'Post' and 'Pre' for better clarity. Thank you for your valuable feedback.

**Reviewer #2: The analysis presented do match the analysis plan.
The results are clearly presented and are original and complete.
Figures/tables are clear and self-explaining.**

A: We appreciate your positive feedback regarding our manuscript. We are pleased to know that the analysis and results were considered original and complete. And we also appreciate your recognition that the figures and tables are clear. Your feedback is extremely valuable to us.

**Conclusions**

- **Are the conclusions supported by the data presented?**
- **Are the limitations of analysis clearly described?**
- **Do the authors discuss how these data can be helpful to advance our understanding of the topic under study?**
- **Is public health relevance addressed?**

**Reviewer #1: (No Response)**

**Reviewer #2: Conclusions are supported by the data presented and are well discussed. The topic of the manuscript is of relevant interest and its public health relevanca is properly addressed.**

A: We would like to thank you for your positive feedback. We are pleased that the conclusions are well supported and the relevance of the topic to public health is clear and addressed.

**Editorial and Data Presentation Modifications?**

**Use this section for editorial suggestions as well as relatively minor modifications of existing data that would enhance clarity. If the only modifications needed are minor and/or editorial, you may wish to recommend “Minor Revision” or “Accept”.

Reviewer #1: Please revise the manuscript to adhere to the journal's standards, including the abstract format and citation style. Additionally, take into account the annotations provided in the manuscript PDF to enhance clarity, particularly in terms of language structure.**

A: Thank you for your suggestions. We appreciate your guidance regarding the abstract format, citation style, and the annotations in the manuscript PDF. We will make sure to address these points thoroughly in our revision. Your feedback is extremely valuable to us and motivates us to continue improving our work.

**Reviewer #2: (No Response)**

**Summary and General Comments**

**Use this section to provide overall comments, discuss strengths/weaknesses of the study, novelty, significance, general execution and scholarship. You may also include additional comments for the author, including concerns about dual publication, research ethics, or publication ethics. If requesting major revision, please articulate the new experiments that are needed.**

**Reviewer #1: The manuscript needs a clear justification for its claim of relative cost-effectiveness, particularly in comparison to other available methods. Specifically, how does the cost of lc WGS compare to alternative approaches? This clarification is essential for assessing the study's practical implications and potential adoption in relevant fields.**

A: We sincerely appreciate your suggestions regarding the justification of our manuscript. We recognize the importance of a clear rationale for the claim of relative cost-effectiveness of lcWGS compared to other available approaches. In our revised version, we have clarified the use of lcWGS in the discussion section of the text, including more details (supported by the references included as 33 and 34) on how the costs of the technic compares to existing alternatives. This point is crucial for assessing the practical implications of the study and its potential adoption in relevant fields. Once again, we thank you for your valuable corrections.

**The map presented in Figure 1 would benefit from including coordinates relative to the North arrow for improved orientation. Furthermore, it's important to ensure compliance with copyright regulations when utilizing data from Google Maps in QGIS. The authors should verify that any data sourced from Google Maps are permissible under the cc-by 4.0 license.**

A: We appreciate the reviewer's valuable suggestions. Coordinates were included on the map in figure 1, as suggested. Additionally, we inform you that the data used in the base maps belongs to the open data platform Open Street Map (OSM), licensed under the Open Data Commons Open Database License (ODbL) by the OpenStreetMap Foundation (OSMF), which gives free access to copy, distribute, transmit and adapt the data (<https://www.openstreetmap.org/copyright>).

**The concept of the bottleneck effect, referenced in both Line 47 and Line 246, is inadequately explained in the context of the population under study. Given its significance, a more thorough elucidation is warranted to ensure clarity and understanding for the reader. This discussion is crucial and should be more prominently addressed within the manuscript.**

A: We thank the reviewer for their valuable suggestions. We have revised the manuscript to provide a more thorough explanation of the bottleneck effect in the context of the population under study. This discussion is now more prominently addressed to ensure clarity and understanding for the reader.

**Reviewer #2: A nice manuscript, well written and with original data that represent a relevant step-forward of the knowledge in the field.**

A: We would like to sincerely thank you for the positive comments regarding our manuscript. We are happy to hear that you found it well-written, original, and a significant advancement in the field.

**PLOS authors have the option to publish the peer review history of their article (**[**https://journals.plos.org/plosntds/s/editorial-and-peer-review-process#loc-peer-review-history**](https://journals.plos.org/plosntds/s/editorial-and-peer-review-process#loc-peer-review-history)**). If published, this will include your full peer review and any attached files.**

**If you choose “no”, your identity will remain anonymous but your review may still be made public.**

**Do you want your identity to be public for this peer review?</b> For information about this choice, including consent withdrawal, please see our "**[**https://www.plos.org/privacy-policy**](https://www.plos.org/privacy-policy)**" Privacy Policy.**

**Reviewer #1: No**

**Reviewer #2: No**
